# Use of sensory processing information in the diagnosis of autism spectrum disorder and attention deficit hyperactivity disorder in children at an Australian community hospital

**DOI:** 10.1111/1440-1630.70007

**Published:** 2025-03-13

**Authors:** Annabelle Marozza, Karen Hay, Thuy Frakking

**Affiliations:** ^1^ Occupational Therapy Department Caboolture Hospital, Metro North Hospital & Health Service Caboolture Queensland Australia; ^2^ Statistics Unit QIMR Berghofer Medical Research Institute Brisbane Queensland Australia; ^3^ Research Development Unit Caboolture Hospital Metro North Hospital & Health Service Caboolture Queensland Australia; ^4^ Child Health Research Centre, Faculty of Medicine The University of Queensland South Brisbane Queensland Australia; ^5^ Speech Pathology Department Gold Coast University Hospital, Gold Coast Hospital & Health Service Southport Queensland Australia; ^6^ School of Health Sciences & Social Work Griffith University Southport Queensland Australia

**Keywords:** assessment, Australia, autism spectrum disorder, diagnosis, sensory processing, attention deficit hyperactivity disorder

## Abstract

**Introduction:**

The provision of sensory processing information is one aspect of occupational therapy involvement in diagnostic assessment for autism spectrum disorder and attention deficit hyperactivity disorder. The aim of this study was to retrospectively compare SHORT Sensory Profile 2 results of children with suspected diagnoses and assess the discriminatory ability of modified scales of SHORT Sensory Profile 2 to identify diagnostic criteria.

**Method:**

This study involved a retrospective chart audit of SHORT Sensory Profile 2 results and paediatrician letter of diagnosis (*N* = 92) for children aged 6–13 years who had multidisciplinary diagnostic assessment through outpatient paediatric service.

**Consumer and Community Involvement:**

No consumers participated in the study design or analysis. Study involved retrospective analysis of de‐identified results from caregiver assessments.

**Results:**

Forty nine per cent of children in study sample had confirmed diagnosis of attention deficit hyperactivity disorder, and 26% had diagnosis of autism spectrum disorder. Average percentage scores for both behavioural and sensory components of the SHORT Sensory Profile 2 were highest amongst children with a diagnosis of autism spectrum disorder, as well as higher classification for avoiding and seeking quadrants. In relation to modified scales, Cronbach's alpha indicated high internal consistency for inattention (0.84) and social communication and interaction (0.86). Based on the ROC analyses, the discriminatory ability when all the modified scales are used in combination ranged from poor (original four quadrants to distinguish ADHD vs no diagnosis: AUC = 0.59) to good (ASD vs ADHD; AUC = 0.82; ASD vs no diagnosis (AUC = 0.81).

**Conclusion:**

Results of this study highlight the need for more rigorous investigation for validation of modified scales and consensus within occupational therapy and multidisciplinary team in relation to reporting of sensory processing information and contribution to diagnostic criteria.

**PLAIN LANGUAGE SUMMARY:**

Many children with attentional difficulties or autism require information from occupational therapists about their sensory issues to help inform and identify behaviours of concern. This study looked at how a sensory processing screening tool can identify traits related to attentional difficulties and autism in children. Seventy‐eight medical charts of children from a community hospital were reviewed. This study showed that information from the sensory processing screening tool related to attention and social communication traits. Clinicians can consider using information from a sensory processing screening tool in combination with other assessments to contribute to the identification of attention difficulties and autism in children.

**Key Points for Occupational Therapy**

- Sensory processing information contributes to differential diagnosis of ASD and ADHD in children.
- Contribution to diagnostic process requires description of the child's presenting behaviours in relation to DSM‐5 criteria.
- Further consensus is required for developing clinical guidelines for the reporting of sensory processing functioning.

## 1 INTRODUCTION

Assessment of sensory processing (SP) is a recommended part of the diagnostic workup for neurodiversity as outlined in the Australian guidelines for the assessment of autism spectrum disorder (ASD) (Goodall et al., 2023) and Australian Evidence Based Clinical Practice Guideline for attention deficit hyperactivity (ADHD) (ADHD Guideline Development Group, 2022). SP assessment is important because it provides an understanding of presenting behaviours that are seen in the clinic or reported in the collateral information from school, parental reports and other multidisciplinary (MDT) assessments. Occupational therapists play a key role in the assessment of sensory processing disorder in children and provide intervention to support children's engagement and participation in activities of daily living, education, leisure and play, and social activities (Ahn et al., 2004; Al‐Heizan et al., 2015).

Sensory processing refers to the neurophysiological processes involving the reception, modulation integration, and organisation of sensory stimuli, including the behavioural responses to sensory input (Miller & Lane, 2000). Sensory modulation is one dimension of SP, defined as difficulty in regulating different sensory inputs and can result in a variety of responses including sensory over responsiveness, sensory under responsiveness and sensory seeking behaviours (Case‐Smith et al., 2015; Libermann et al., 2020). The SHORT Sensory Profile 2 (SHORT SP2) is a 34‐item screening tool designed to assess SP in children aged between 3:0 years and 14:11 years (Dunn, 2014). It was developed from the CHILD Sensory Profile 2 (2014). The tool is based on Dunn's Sensory Processing Framework (Dunn, 2014) that conceptualises SP contributions to a child's behaviour (Brown & Dunn, 2010). The model explores two factors in relation to response to sensory stimuli, including neurological threshold and self‐regulation continuum (Jorquera‐Cabera et al., 2017). The relationship between the two factors can indicate one of four SP patterns referred to as quadrants on the sensory profile tool, including sensory seeking, sensory avoiding, sensory registration and sensory sensitivity. Item responses are based on the frequency of the behaviour occurring ranging from score of 1 (*almost never* = 10% time or less) to 5 (*almost always* = 90% time or more). Summary scores for sensory items and behaviour items can be calculated, and when interpreting summary scores, the use of the terms ‘more than others’ and ‘less than others’ informs the child's engagement in behaviours in comparison to peers (Dunn, 2014). The SHORT SP2 has discriminative validity of over 95% in differentiating between children with and without sensory modulation differences (Tomchek & Dunn, 2007). SHORT SP2 discriminates in a similar way to the CHILD Sensory Profile 2 (Dunn, 2014). This tool is the second most used measure for ASD (Burns et al., 2017) and has been used by Galiana et al. (2022) to explore sensory processing difficulties in school age children from Spain. The prevalence of SP differences is reported to be 95% for children who are on the autism spectrum (Tomchek & Dunn, 2007). Differences in sensory processing based on parental report for children who have ADHD traits are reported in several studies (Dunn & Bennett, 2002; Kalpogianni, 2002; Yochman et al., 2004).

During the diagnostic process, health and medical professionals refer to the Diagnostic and Statistical Manual of Mental Disorders (5th ed; DSM‐5; American Psychiatric Association, 2022) (American Psychiatric Association [APA], 2022) and The International Statistical Classification of Diseases and related health conditions 11th edition (ICD‐11; World Health Organisation) (World Health Organisation [WHO], 2022). Diagnostic criteria for ADHD include the presence of six of the nine criteria related to inattention and hyperactivity–impulsivity before 12 years of age and across more than one setting, such as school and home. Assessment of symptoms facilitates classification of ADHD presentations as inattentive, hyperactive–impulsive or combined where both inattentive and hyperactive–impulsive presentations are present. The DSM‐V criteria for ASD includes severity rating across the two patterns of behaviour associated with autism spectrum: (a) social communication and social interaction and (b) restricted and repetitive behaviour, interests or activities. Other criteria include behaviours being present in the early developmental period and resulting in significant impairments in social, occupational and other important areas of current functioning (American Psychiatric Association, 2022). National practice guidelines (ADHD Guideline Development Group, 2022; Goodall et al., 2023) provide guidance on how MDT teams can use these diagnostic systems in clinical practice and enhance consensus. The use of the SHORT SP2 has demonstrated the relationship with sensory seeking and sensitivity to predict inattention and hyperactivity–impulsivity (Delgado‐Lobete et al., 2020). Sensory difficulties in children with ASD have been related to restricted, repetitive behaviours and restricted interests (Burns et al., 2017).

ADHD is reported as the most common mental health disorder in Australian children aged 4–17 years (Lawerence et al., 2015) and prevalence in Australian children and adolescents is estimated to be 1.2% children aged 0–14 years in Australia (Sincovich et al., 2020). Children with ADHD traits may present with a range of SP patterns including sensory avoiding (when they are more bothered by sensory information), sensory sensitive (when they are more aware of sensory information) and low registration (having less detection of sensory information) (Delgado‐Lobete et al., 2020). Many children with ADHD have differences in sensory areas including auditory (Ghanizadeh, 2009) olfactory, tactile (Mangeot et al., 2001), vestibular and visual sensory systems (Jung et al., 2014), proprioceptive and vestibular processing (Ghanizadeh, 2011).

Criteria associated with ASD can be present before 3 years of age but are often not apparent until school years when social and cognitive demands increase. The co‐occurrence of language and learning difficulties and a range of medical conditions is common (Goodall et al., 2023). The prevalence of ASD in Australian children is reported as 2.1% for children aged 0–14 years and ASD has been indicated as the leading cause of burden of disease in boys aged 5–14 years and the fourth cause in girls (Australian Institue of Health and Welfare [AIHW], 2024). Studies have demonstrated that children with ASD have increased sensitivities, avoidance, and sensory seeking behaviours compared with children with typical development (Baranek et al., 2006; Ben‐Sasson et al., 2009; Rodgers & Ozonoff, 2005). Children with ASD have impairments with auditory, visual and tactile processing skills (Simpson et al., 2019), and hyper‐sensitivities where there is limited tolerance for the amount and intensity of sensory input.

Characterising children based on SP subtypes adds to understanding how SP patterns impact behaviour (Little et al., 2017). The subscale scores from an earlier version of the SHORT Sensory Profile (1999) demonstrated variability in sensory responses for children with ASD (Lane et al., 2011; Tomchek & Dunn, 2007; Uljarevic et al., 2016). A more recent study using the SHORT SP2 identified two predominant subtypes in children with ASD including (a) children with uniformly elevated profile (i.e., high scored across all sensory profile quadrants) and (b) children with raised avoiding and sensitivity profile (i.e., raised scores in the avoiding and sensitivity quadrants) (Simpson et al., 2019). Other studies have used both item and subscale data to compare sensory responses across disabilities (Burns et al., 2017; Green et al., 2016; Rodgers et al., 2003; Tomchek & Dunn, 2007). Little et al. (2017) described five subtype model that compared the sensory profiles of children with ASD, ADHD, learning disabilities and typically developing children. One of the subtypes identified was a balanced sensory profile for children with ASD (35.1%) and ADHD (53.1%) (Little et al., 2017). The balanced sensory profile subtype indicates evenly distributed sensory profile quadrants. This issue of responses across all SP quadrants is a common interpretation challenge faced by occupational therapists in the reporting the results of sensory profiles that has been addressed in the revised edition of the sensory profile tools, where a strength perspective is promoted with the interpretation of a child's sensory profile to consider the pattern of scores across all of the four quadrants (Dunn, 2014).

The SHORT SP2 is based on caregiver report, completed face to face, online or via telephone (DuBois et al., 2017). The flexibility of administrative modalities is applicable for modern healthcare due to (a) its reduced screening time and report writing time, which helps to improve efficiencies within an increasingly burdened public health system, and (b) its ease of administration via phone or telehealth, (c) reducing burden on caregivers who are completing several questionnaires during the diagnostic process. Best practice guidelines recommend that the assessment of SP should be completed within natural contexts to assist with diagnostic and interventional support (Donaldson et al., 2017; Dunn, 1999). However, sensory checklists within public health system are completed within clinical settings. The interpretation of SP results is variable due to limited guidelines regarding reporting of results especially within an MDT service model.

Australian studies have identified that over 90% of parents accessed some form of professional help for their child's behaviour or learning prior to their formal diagnosis (Hiscock et al., 2017). There is an increasing reliance on the public health system which often involves long wait times and depending on staffing resourcing, there may be limited MDT consultation. The administration of specific diagnostic tools for ASD requires specific staff certification, which may be another barrier in accessing diagnostic services. The information provided from SP assessment needs to be easily interpreted in the context of functional impairments and related to the diagnostic criteria for the identification of ASD and/or ADHD traits. To contribute to the diagnosis process, occupational therapists can use the results of a child's individual sensory profile to demonstrate the functional and behavioural impact in relation to the DSM‐5 criteria.

Based on clinical experience, we postulated that modified scales derived from items from the SHORT SP 2 may be useful in identifying relevant DSM‐5 criteria and hence the diagnosis of ASD and ADHD.

The aims of the study re

1. to describe and compare original SHORT SP2 quadrant and section scores and proposed modified scale scores for children diagnosed with ASD or ADHD;
2. to assess the internal consistency of the proposed modified scales for measuring the latent constructs of inattention, hyperactivity, social communication and repetitive behaviours;
3. to compare modified scale scores by report of relevant DSM‐5 criteria; and
4. to assess the discriminatory ability of the modified scales and SHORT SP 2 quadrant scores in correctly classifying children by diagnosis of ASD, ADHD or no diagnosis.

## 2 METHODS

### 2.1 Study design

A retrospective observational cohort study was conducted at Caboolture Hospital paediatric allied health diagnostic outpatient clinic in Queensland, Australia. Ethics approval was obtained from The Prince Charles Hospital, Queensland (HREC/2021/QCPH/80564).

### 2.2 Inclusion and exclusion criteria

**FIGURE 1:**
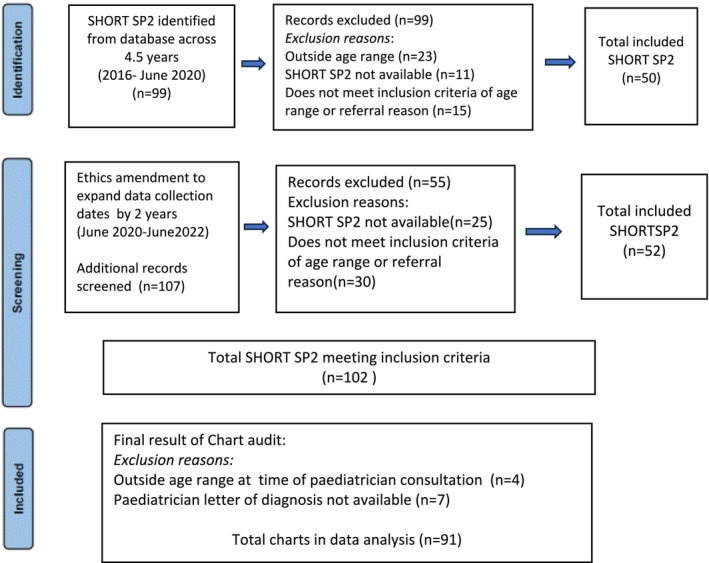
Chart audit screening process.

Children aged 6–13 years who had a SHORT SP2 assessment administered as part of a diagnostic workout for suspected ASD or ADHD between January 2016 and June 2022 and a final letter of diagnosis by the paediatrician were eligible for inclusion. Children were excluded if SHORT SP2 (Dunn, 2014) was not completed or if the SHORT SP2 results were missing from the MDT report. The SHORT SP2 results were not available if assessment had been conducted by a private occupational therapy service in the community. A final letter of diagnosis was not available if the child did not attend the paediatrician consultation. Figure 1 outlines the chart audit screening process.

The SHORT SP2 is one assessment completed by parent and/or caregiver during occupational therapy assessment sessions. The diagnostic clinic produces one MDT report, with individual discipline clinicians completing their relevant sections of the report. An experienced occupational therapist identified cluster items to derive modified scales to assess specific DSM‐5 criteria used to diagnose ASD and ADHD. The cluster items were identified based on clinical experience and the items most referenced by the clinician in her assessment reporting. The clinician reports item results from SHORT SP2 related to the DSM‐5 criteria: inattention (IN), hyperactivity–impulsivity (HI), social communication and interaction (SCI) and repetitive or restricted patterns of behaviour (RRI). Refer to Data S1 for the list of modified scales.

### 2.3 Data collection

Baseline characteristics extracted from the electronic medical records comprised age, gender and comorbidities. Individual item scores were extracted from SHORT SP2 forms and overall quadrant (Seeking, Avoiding, Sensitivity and Registration) and section (Sensory and Behavioural) scores with corresponding categories (much less than others, less than others, just like the majority of others and more were derived. The definitive diagnosis was determined from review of the paediatric letter of diagnosis. Data extracted included the documented diagnosis (ADHD/ASD/other/no diagnosis), SHORT SP2 results (yes/no), sensory preferences (seeking, avoiding, sensitivity and registration). Criteria related to DSM‐5 included in the paediatrician letter of diagnosis, any of the following words: inattention, hyperactivity, impulsivity, social communication, social interaction, repetitive behaviour and restricted interests. If DSM‐5 criteria were not included in paediatrician letter of diagnosis, then it was assumed the criteria was not present.

### 2.4 Data analysis

Children were classified into three groups of interest (ASD, ADHD or no diagnosis) based on the diagnosis recorded in the paediatrician's letter of diagnosis. Children with both ADHD and ASD or other diagnoses as documented in paediatrician letter of diagnosis, were excluded from analyses. Percentage scores were derived for each of the original SHORT SP2 quadrants, sensory and behavioural sections, and modified scales. Quadrant and section scores were classified using the published cut‐points for the normal distribution (Dunn, 2014).

Categorical variables were summarised as frequency (%) and compared between groups using Pearson's chi‐square test or Fisher's exact test (if >20% cells had <5 observations). Continuous approximately normally distributed variables were summarised as mean (SD) and compared between groups using independent sample *t*‐tests. Baseline characteristics, quadrant sores and modified scale scores were compared by diagnosis (ASD/ADHD). Modified scales scores were compared by report of the relevant DSM‐5 criteria. Cronbach's alpha was estimated as a measure of internal consistency to assess whether the selected items for each of the proposed modified scales were sufficiently similar to other items in the scale.

Parametric receiver operating curve (ROC) analyses were used to assess the discriminatory ability of the scales (individually and in combination) in differentiating the diagnoses of ASD and ADHD compared with each other and no diagnosis. Analyses were performed using the Stata statistical software package (Version 15).

### 2.5 Positionality statement of authors

First and third authors are experienced in diagnostic assessments and paediatric clinical experience and have previously worked (third author) or currently working (first author) with multidisciplinary team service which this study was conducted. Second and third authors hold advanced higher education at PhD level and have extensive research experience. Second author is experienced biostatistician and conducted all data analysis. First author is certified in administration of autism spectrum disorder assessment and has current clinical experience with the national diagnostic guidelines. Collaboration as a team has been integral to the study conceptualisation and understanding of the data.

## 3 RESULTS

### 3.1 Study demographics

Of the 98 children in the age range of interest and referred for diagnostic workup for possible ADHD or ASD, 91 had both SHORT SP2 assessment information and a paediatric letter of diagnosis. One with a dual diagnosis of ASD and ADHD and 12 with other diagnoses and were excluded from analyses. Excluded diagnoses included speech and language disorder (4), intellectual impairment (2) and anxiety (6). Of the 78 included, 38 (49%) diagnosed with ADHD, 20 (26%) with ASD and 20 with no diagnosis were included in analyses (Table 1). The no diagnosis group included conditions that do not have DSM‐5 criteria. This included delayed cognition (6), learning difficulties (4), trauma (4) and behavioural issues (6).

**TABLE 1:** Distribution of variables of interest by final diagnosis of paediatrician.

Variable	Category/measure	Total	No diagnosis	ADHD	ASD	*p* value
		*N* = 78	*N* = 20	*N* = 38	*N* = 20	
Age^a^ (years)		8.9 (2.1)	8.4 (1.9)	8.9 (2.1)	9.4 (2.4)	0.36
Gender^b^	Male	54 (69%)	16 (80%)	28 (74%)	10 (50%)	0.071
	Female	24 (31%)	4 (20%)	10 (26%)	10 (50%)	
DSM‐5 criteria reported^c^	*N* (%)					
Inattention		24 (31%)	4 (20%)	18 (47%)	2 (10%)	
Hyperactivity		24 (31%)	5 (25%)	18 (47%)	1 (5%)	
Restricted interest/repetition		7 (9%)	0 (0%)	0 (0%)	7 (35%)	
Social/communication issues		13 (17%)	2 (10%)	2 (5.3%)	9 (45%)	
Proposed new scales (%)	Mean score % (SD)					
Inattention (IN)		64 (18)	55 (22)	66 (14)	68 (19)	0.59
Hyperactivity (HI)		61 (25)	53 (19)	64 (25)	65 (30)	0.95
Restricted interest/repetition (RRI)		60 (22)	53 (21)	57 (20)	71 (20)	<0.001
Social/communication (SCI)		54 (19)	48 (18)	50 (18)	68 (17)	0.017
SHORT SP2 components^d^	Mean score % (SD)					
Behavioural		64 (18)	55 (20)	64 (15)	72 (19)	0.087
Sensory		56 (18)	49 (18)	54 (17)	66 (18)	0.011
SHORT SP2 quadrants^d^ (%)						
Seeking		61 (20)	55 (16)	61 (21)	68 (21)	0.27
Sensitivity		63 (20)	55 (21)	62 (18)	72 (19)	0.064
Registration		47 (22)	38 (23)	46 (19)	58 (23)	0.038
Avoiding		69 (19)	62 (24)	68 (15)	78 (17)	0.030
SHORT SP2 classification (*N*, %)						
Behavioural classification	Like majority	70 (90%)	19 (95%)	35 (92%)	16 (80%)	0.17
	More than others	7 (9%)	1 (5%)	2 (5.3%)	4 (20%)	
	Much more than others	1 (1.3%)	0 (0%)	1 (2.6%)	0 (0%)	
Sensory classification	Like majority	75 (96%)	20 (100%)	37 (97%)	18 (90%)	0.23
	More than others	3 (3.8%)	0 (0%)	1 (2.6%)	2 (10%)	
Seeking classification	Like majority	68 (87%)	20 (100%)	32 (84%)	16 (80%)	0.47
	More than others	7 (9%)	0 (0%)	5 (13%)	2 (10%)	
	Much more than others	3 (3.8%)	0 (0%)	1 (2.6%)	2 (10%)	
Sensitivity classification	Like majority	69 (88%)	19 (95%)	35 (92%)	15 (75%)	0.073
	More than others	9 (12%)	1 (5%)	3 (7.9%)	5 (25%)	
Registration classification	Less than others	3 (3.8%)	2 (10%)	0 (0%)	1 (5%)	0.073
	Like majority	71 (91%)	18 (90%)	37 (97%)	16 (80%)	
	More than others	4 (5.1%)	0 (0%)	1 (2.6%)	3 (15%)	
Avoiding classification	Like majority	64 (82%)	18 (90%)	34 (89%)	12 (60%)	0.029
	More than others	9 (12%)	2 (10%)	2 (5.3%)	5 (25%)	
	Much more than others	5 (6.4%)	0 (0%)	2 (5.3%)	3 (15%)	

*Note*: *p* values relate to contrast between ADHD and ASD.

Abbreviations: ADHD, attention deficit hyperactivity disorder; ASD, autism spectrum disorder; DSM‐V criteria reported (in paediatrician letter of diagnosis) documentation of words IN, Inattention; HI, hyperactivity–impulsivity; RRI, repetitive behaviour/restricted interest; SCI, social communication and interaction.

^a^ Mean (SD) with *p* value from independent *t*‐test.

^b^ Number (%) with *p* values from Pearson's chi‐square test.

^c^ Fisher's exact test.

^d^ Age in years.

The mean age was 8.9 (SD 2.1) years and 69% were male. Of the DSM‐5 diagnostic criteria of interest, inattention and hyperactivity were each reported in 24/78 (31%) children overall and in 18/38 (47%) children with a diagnosis of ADHD, inclusive of 12 with both criteria reported. For 14/38 (37%) no documentation of the specific DSM‐5 criteria identified for this study was included in the paediatrician's letter of diagnosis. Amongst children diagnosed with ASD, RRI was documented in 7/20 (35%) children diagnosed with ASD and SCI was documented in 9/20 (45%); no documentation of the specific DSM‐5 criteria identified for this study were documented for 7/20 (35%).

Mean percentage scores for the modified scales for RRI (71% vs 57%; *p* < 0.001) and SCI (68% vs 54%; *p* = 0.017) were significantly higher in children with ASD compared with those with ADHD (Table 1). The mean percentage scores for the sensory component of the SSP2 (66% vs 54% *p* = 0.011) and for the Registration and Avoiding quadrants were also significantly higher in children with a diagnosis of ASD compared children with ADHD. After classification using recommended cut‐point from normative data, the majority of children were classified ‘like majority’ across all quadrants. Of children with ASD, 15% were classified as ‘much more than others’ in the avoidant quadrant and 10% were classified as ‘much more than others’ in the seeking quadrant.

### 3.2 Internal consistency of modified scales

In our sample, Cronbach's alpha indicated high internal consistency for the modified scales for IN (0.84) and SCI (0.86) and acceptable internal consistency for RRI (0.76) and HI (0.73) (Table 2).

**TABLE 2:** Internal consistency of modified scales to DSM‐5 criteria.

Construct	Measure	Item –test correlation	Item‐rest correlation	Average inter‐item covariance	Cronbach's alpha
Inattention (IN)	IN scale	0.52–0.68	0.41–0.62	0.44–0.51	0.82–0.83
	11 items (range)			0.47	0.84
Social/communication (SCI)	SCI scale	0.52–0.81	0.42–0.66	0.62–0.72	0.83–0.85
	10 items (range)			0.68	0.86
Repetitive/restricted interest (RRI)	RRI scale	0.44–0.73	0.29–0.61	0.44–0.54	0.70–0.76
	9 items (range)			0.49	0.76
Hyperactivity–impulsivity (HI)	HI scale	0.70–0.76	0.47–0.60	0.75–0.89	0.63–0.69
	4 items (range)			0.81	0.73

Abbreviations: IN, Inattention; SCI, social communication and interaction; RRI, repetitive behaviour/restricted interest; HI, hyperactivity–impulsivity.

### 3.3 Modified scales by DSM‐5 criteria

The mean percentage scores for the RRI scale were 74% vs 52% in those with/without the DSM‐5 RRI criterion reported (*p* = 0.004) while the mean HI scale scores were 70% vs 58% in those with/without the DSM‐5 HI criterion reported (*p* = 0.059) (Table 3). There was no evidence for differences in mean percentage scores for IN or SCI by report of the relevant DSM‐5 criteria.

**TABLE 3:** Comparison of mean percentage scores for modified scales by report of DSM‐5 diagnostic criteria in the paediatrician's letter of diagnosis.

DSM‐5 criteria	Reported	Modified scale	Criterion absent	Criterion present	*p* value^a^
	*n* (%)		Mean (SD)	Mean (SD)	
Inattention	24 (31%)	Inattention (IN)	64 (19)	64 (16)	0.99
Hyperactivity–impulsivity	24 (31%)	Hyperactivity–impulsivity (HI)	58 (26)	70 (23)	0.059
Repetitive/restricted interest	7 (9%)	Repetitive/restricted interest (RRI)	52 (18)	74 (22)	0.004
Social/communication	13 (17%)	Social/communication (SCI)	59 (21)	63 (25)	0.53

^a^ *p* value derived from independent sample *t*‐test.

### 3.4 Discriminatory ability of modified scales in diagnosing ASD or ADHD

Based on areas under the ROC curve (AUC) (Table 4), the discriminatory ability of the original scales and the modified scales as a single predictor in diagnosing ADHD or ASD ranged from nil (e.g., HI for ADHD vs ASD: AUC = 0.50) to moderate. None of the scales provided acceptable discrimination between ADHD and no diagnosis in this sample. Moderate discrimination was observed for the proposed RRI (AUC: 0.80) and SCI (AUC: 0.74) scales in discriminating between ASD and no diagnosis. When all scales were used in combination, discriminatory ability ranged from poor (original four quadrants to distinguish ADHD vs no diagnosis: AUC = 0.59) to good (ASD vs ADHD; AUC = 0.82; ASD vs no diagnosis (AUC = 0.81).

**TABLE 4:** Comparison of discriminatory ability of modified scales and original quadrant scores derived from the short SP2 tool in correctly classifying children by diagnosis.

	Classification		
Scale	ADHD vs no diagnosis (*n* = 60)	ASD vs no diagnosis (*n* = 40)	ADHD vs ASD (*n* = 58)
	AUC (95% CI)	AUC (95% CI)	AUC (95% CI)
**Modified scales**			
Inattention	0.66 (0.51–0.82)	0.68 (0.52–0.84)	0.54 (0.38–0.7)
Social/communication	0.56 (0.41–0.72)	0.74 (0.59–0.89)	0.69 (0.55–0.83)
Repetitive/restricted interest	0.53 (0.38–0.69)	0.80 (0.67–0.93)	0.77 (0.65–0.90)
Hyperactivity–impulsivity	0.64 (0.5–0.78)	0.63 (0.47–0.80)	0.50 (0.35–0.66)
All combined	0.71 (0.55–0.86)	0.81 (0.67–0.95)	0.82 (0.72–0.93)
**SHORT SP2 scales**			
Seeking	0.60 (0.46–0.74)	0.70 (0.54–0.86)	0.59 (0.44–0.74)
Sensitivity	0.61 (0.46–0.76)	0.73 (0.58–0.88)	0.64 (0.50–0.79)
Registration	0.60 (0.45–0.76)	0.73 (0.58–0.88)	0.66 (0.51–0.81)
Avoiding	0.59 (0.43–0.75)	0.71 (0.55–0.87)	0.67 (0.52–0.81)
All quadrants combined	0.59 (0.42–0.76)	0.76 (0.61–0.91)	0.75 (0.6–0.89)

Abbreviations: ADHD, attention deficit hyperactivity disorder; ASD, autism spectrum disorder; IN, Inattention; SCI, social communication and interaction; RRI, repetitive behaviour/restricted interest; HI, hyperactivity–impulsivity

## 4 DISCUSSION

In this retrospective study we have explored the utility of using modified scales based on cluster items of the SHORT SP2, to identify the DSM‐5 criteria for ADHD and ASD. Concise documentation of sensory processing assessment results related to DSM‐5 criteria was needed within the study service model as the MDT report was the only mode of communication with the medical staff. Ensuring effectiveness of written documentation of sensory processing assessment results, is relevant within current clinical practice based on the recommendations within The Australian Guidelines for Assessment of ASD (Goodall et al., 2023) in relation to providing information which is comprehensive and understandable to the client. The Australian Evidence Based Clinical Practice Guidelines for ADHD (ADHD Guideline Development Group, 2022) recommends the need to provide information to educate people about the symptoms and functional impact of ADHD.

It has been reported that the average age for ADHD diagnosis is between 5 and 9 years and between 3 and 6 years for ASD (Knott et al., 2024). In our study, the mean age at diagnosis for was 8.9 years for the ADHD group and 9.2 years for the ASD group, consistent with the work of Boulton et al. (2023) who identified an average delay of 3.5 years for diagnostic assessment in Australia. Our study sample had a higher percentage of confirmed diagnosis from paediatrician letter of ADHD (47%) compared with ASD (26%). Referrals to the service come from community based general practitioners and are for complex behavioural issues. The higher rate of confirmed ADHD diagnosis in our study sample may be reflective of externalised behaviours such as hyperactivity and attentional difficulties identified by schools. Due to disruption on a child's participation within the school, parents are encouraged to seek specialist consultation, leading to the initiation of the diagnostic process.

In our sample study, the one documented co‐occurring ASD and ADHD case was not included. In cases where collateral information indicated a range of sensory processing difficulties and the possibility of co‐occurrence of ASD and ADHD, the occupational therapist would have chosen to administer the CHILD Sensory Profile 2 (Dunn, 2014) rather than screener version of SHORT SP2. In relation to diagnosis delay where there is a co‐occurrence of ASD and ADHD it has been found that ASD tends to be diagnosed at a later age when ADHD is present and ADHD is diagnosed at an earlier age when ASD is present (Sainsbury et al., 2023). The recent guidelines for assessment of ASD and evidence based clinical practice guidelines for ADHD both recommend the screening of co ‐occurring conditions. Reporting the results from SHORT SP2 using the modified scales could provide time and resource efficiency for MDT service models in the reporting of traits of ASD and/or ADHD the child may be presenting with.

The classification system of the SHORT SP2 is based on normative data and 2% of the population are expected to score within ‘much more than others ‘range (Dunn, 2014). Comparison of results between studies that have used SHORT SP2 is challenging due to differences in sample characteristics, age ranges and differences in reporting of quadrant scores. Simpson et al., 2019 reported ‘much more than others’ classification for children with ASD for 62.1% in the avoiding quadrant and 65.7% in the sensitivity quadrant. By contrast, 15% and 10% of children with ASD in our study were classified as ‘much more than others’ for avoiding and seeking quadrants, respectively. It is important to note that Simpson study had a wider age range of 4 years to 11 years 5 months. Delgado‐Lobete et al. (2020) reported a higher prevalence of sensitivity processing patterns (77.8%), followed by seeking patterns (66.7%) based on classification of total quadrant scores in children with ADHD. Of children with ADHD in our study sample, seeking processing pattern was most common, with more than others (13%) and much more than others (2.6%) responses. These results reflect the variability of sensory processing patterns in children with ADHD.

Reporting of sensory processing responses may not be widely understood by members of the MDT and medical team, particularly if they are not familiar with the Dunn's Sensory Processing Framework (Dunn, 2014). The use of the modified scales to measure diagnostic criteria may help with team consensus for diagnosis formulation. Our study results indicated comparison of mean percentage scores for the modified scales of RRI and SCI were significantly higher in children in ASD group compared with those in ADHD group. The use of the modified scales could be used to screen cases where further diagnostic assessment tools, such as Autism Diagnostic Observation Schedule 2 (Lord et al., 2012) would be recommended to verify diagnosis of ASD.

When considering the internal consistency of the modified scales, Cronbach's alpha indicated high internal consistency for inattention (IN) (0.84) and social communication and interaction (SCI) (0.86). A modified scale which more clearly links items from the SHORT SP2 with the DSM‐5 diagnostic criteria would be useful where these behaviours may not be observed within the time restrictions of a clinic setting. As part of MDT diagnostic process, school information outlining concerning behaviours of the child within the school context is obtained. The relevant items from SHORT SSP 2 provide parental report of the child's performance within the home context. The combination of this assessment information provides contextual information further informing the diagnostic process for ADHD and ASD in children.

In relation to DSM‐5 criteria, repetitive and restricted interest (RRI) and hyperactivity (HI) was more commonly identified in those with a confirmed diagnosis. Reporting the specific item wording in combination with sensory profile scoring can provide information not collated by other MDT assessments. For example, in RRI scale, item (11) *shows a strong preference for certain tastes* and item (30) *seems oblivious within an active environment*. In relation to HI scale item (6) *touches people and objects more than same aged peers* and item (32) *jumps from one activity to another so that it interferes with activities*. This information provides functional impact of the traits the child is presenting with. Reporting assessment findings in this way is relevant to the current recommendations of assessment of functioning (Goodall et al., 2023) and documentation of the child's occupational functioning (ADHD Guideline Development Group, 2022).

In this study sample the no diagnosis group (those without DSM‐5 defined diagnosis) was used as a comparison group to assess discriminatory ability. This group included a range of clinical presentations such as delayed cognition, learning difficulties, trauma and behavioural issues—all of which may have had sensory processing difficulties. The RRI AUC (0.80) and SCI (0.74) scales had moderate discriminative ability between ASD and no diagnosis. In clinical settings where staff are not certified in the standardised assessments for ASD, reporting the results from SHORT SP2 using the modified scales may be useful to identify diagnostic traits of ASD and inform the need for further diagnostic assessment related to ASD.

The use of the SHORT SP 2 in diagnostic assessment may not be preferred by some as it is a parent/caregiver report and MDT need to be aware of bias related to parental reporting. This bias may be present in relation to the parents' own experience of depression or anxiety, and parents' knowledge of symptoms associated with diagnoses. Alternatively, the use of the SHORT SP2 may be preferred as it identifies the individual child's sensory processing experiences. Reporting of individuals' experiences is a principle of the neurodiversity paradigm (Izuno‐Garcia et al., 2023), where individual's developmental differences are supported, and individual strengths are recognised. Current practice guidelines do identify the importance of clinical interview to collate all aspects of child's developmental history, onset, severity and the functional impacts of the traits for ADHD (ADHD Guideline Development Group, 2022) and comprehensive needs assessment (Goodall et al., 2023), which involves the use of several assessments rather than the reliance of the results from one assessment.

### 4.1 Limitations

This was an exploratory study investigating existing and proposed modified scales of SHORT SP2 in relation to DSM‐5 criteria for ASD and ADHD diagnosis. The formulation of the modified scales was based on the principal investigator's clinical experiences and related to paediatric population within a public health MDT assessment service. Consensus within occupational therapy and/or MDT groups could improve selection of cluster items included in modified scales. There may have been misclassification bias due to measurement error as there was an assumption that behaviours were absent if DSM‐5 criteria were not reported in the paediatrician letter of diagnosis. It is difficult to generalise the study results to a broader paediatric population due to the small sample size and selected sample characteristics. Further research including expert consensus about grouping of cluster items, assessment in samples with a broader range of clinical presentations and a sufficiently powered prospective validation study incorporating factor analysis of the modified scales are needed to evaluate clinical utility. Hence, the results from this study are exploratory and not conclusive.

## 5 CONCLUSION

This study showed reporting the results from SHORT SP2 using modified scales can identifying repetitive behaviours and restricted interest, and social communication and interaction DSM‐5 criteria related to ASD for the study sample. The grouping of the assessment items for the modified scales indicates high internal consistency for inattention and social communication and interaction. Written documentation reporting sensory processing differences using the modified scales and specific item descriptions, when combined with other MDT assessment information can assist MDT and medical staff in recognition of the child's individual sensory differences and contribute to the collation of diagnostic criteria for ASD and ADHD.

## AUTHOR CONTRIBUTIONS

All authors revised, approved and are accountable for the final manuscript. AM study conceptualisation, methodology and retrospective data collection, writing‐ original draft, review, and editing; KH: contributed to formal analysis and writing review and editing; TF : contributed to study conceptualisation, formal analysis and writing review and editing.

## CONFLICT OF INTEREST STATEMENT

The authors declare that they have no competing interests. This manuscript has not been previously published nor peer‐ reviewed and is not under review elsewhere.

## Supporting information


**Table S1.** SHORT Sensory Profile 2 modified scales diagnosis of ADHD
**Table S2.** SHORT Sensory Profile 2 modified scales diagnosis of ASD

## Data Availability

The data that support the findings of this study are available from the corresponding author upon reasonable request.
