# Supplementary material for: Use of sensory processing information in the diagnosis of autism spectrum disorder and attention deficit hyperactivity disorder in children at an Australian community hospital
Source: Aust Occup Ther J. 2025 Mar 13;72(2):e70007. doi: 10.1111/1440-1630.70007 (PMC11906904; doi:10.1111/1440-1630.70007)
Supplement: Supplementary file 1 — Table S1. SHORT Sensory Profile 2 modified scales diagnosis of ADHD Table S2. SHORT Sensory Profile 2 modified scales diagnosis of ASD [file AOT-72-0-s001.docx]

**Data S1 Supporting Information**

**Table1: SHORT Sensory Profile 2 modified scales diagnosis of ADHD**

| **DSM-5 criteria:** A persistent pattern of inattention and/or hyperactivity-impulsivity that interferes with functioning or development. | | |
| --- | --- | --- |
| ***Total section scores*** | ***Item*** | ***Total score*** |
| Quadrants | Seeking/Seeker | Raw score total/35 |
|  | Avoiding/ Avoider | Raw score total/45 |
|  | Sensitivity/Sensor | Raw score total/50 |
|  | Registration/Bystander | Raw score total/40 |
| Sensory and Behavioural sections | Sensory | Raw score total/70 |
|  | Behavioural | Raw score total/40 |
| **(1) Inattention** | | |
| ***Item section scores*** | ***Item Number*** | ***SHORT Sensory profile statement*** |
| Sensory processing | 2 | Is distracted when there is a lot of noise around |
|  | 9 | Loses balance unexpectedly when walking on uneven surface |
|  | 10 | Bumps into things, failing to notice objects or people in the way |
| Behavioural responses associated with sensory processing | 15 | Seems accident prone |
|  | 19 | Needs positive support to return to challenging situations |
|  | 22 | Gets frustrated easily |
|  | 28 | Struggles to pay attention |
|  | 29 | Looks away from tasks to notice all actions in the room |
|  | 31 | Watches everyone when they move around the room |
|  | 33 | Gets lost easily |
|  | 34 | Has a hard time finding objects in competing backgrounds (for example, shoes in a messy room, pencil in “Junk drawer” |
| **(2) Hyperactivity - impulsivity** | | |
| ***Item section scores*** | ***Item Number*** | ***SHORT Sensory Profile statement*** |
| Sensory processing | 6 | Touches people and objects more than same -aged children |
|  | 7 | Pursues movement to the point it interferes with daily routines (for example, can’t sit still, fidgets) |
|  | 8 | Rocks in chair, on the floor or while standing |
|  | 32 | Jumps from one thing to another so that it interferes with activities |

**Table 2: SHORT Sensory Profile 2 modified scales diagnosis of ASD**

| **DSM-5 criteria: Group of neurodevelopmental disorders characterised by persistent deficits in social communication and social interaction, and by repetitive patterns of behaviour and restricted interests.** | | |
| --- | --- | --- |
| ***Total section scores*** | ***Item*** | ***Total score*** |
| Quadrants | Seeking/Seeker | Raw score total/35 |
|  | Avoiding/ Avoider | Raw score total/45 |
|  | Sensitivity/Sensor | Raw score total/50 |
|  | Registration/Bystander | Raw score total/40 |
| Sensory and Behavioural sections | Sensory | Raw score total/70 |
|  | Behavioural | Raw score total/40 |
| **(1) Social communication and social interaction** | | |
| ***Item section scores*** | ***Item Number*** | ***SHORT Sensory profile statement*** |
| Sensory processing | 3 | Tunes out or seems to ignore me |
|  | 5 | Becomes anxious when standing close to others (for example in a line) |
|  | 14 | Drapes self over furniture or on other people |
| Behavioural responses associated with sensory processing | 17 | Has temper tantrums |
|  | 18 | Resists eye contact from me |
|  | 20 | Has strong emotional outbursts when unable to complete a task |
|  | 21 | Struggle to interpret body language or facial expressions |
|  | 25 | Needs more protection from life than same -aged children (for example, defenceless physically or emotionally) |
|  | 26 | Interacts or participates in groups less than same aged children |
|  | 27 | Misses eye contact with me during everyday interactions |
| **(2) Repetitive patterns of behaviour and restricted interests** | | |
| ***Item section scores*** | ***Item Number*** | ***SHORT Sensory Profile statement*** |
| Sensory processing | 1 | Struggles to complete tasks when music or TV is on |
|  | 4 | Shows distress during grooming (for example, fights or cries during haircutting, face washing, fingernail cutting) |
|  | 11 | Shows a strong preference for certain tastes |
|  | 12 | Moves stiffly |
|  | 13 | Becomes tired easily, especially when standing or holding the body in one position |
| Behavioural responses associated with sensory processing | 16 | Can be stubborn and uncooperative |
|  | 23 | Has fears that interferes with daily routines |
|  | 24 | Is distressed by changes in plans, routines or expectations |
|  | 30 | Seems oblivious within an active environment (for example unaware of activity) |
